# Temperature-Dependent Development Models Describing the Effects of Temperature on the Development of the Fall Armyworm *Spodoptera frugiperda* (J. E. Smith) (Lepidoptera: Noctuidae)

**DOI:** 10.3390/insects13121084

**Published:** 2022-11-24

**Authors:** Matabaro Joseph Malekera, Rajendra Acharya, Md Munir Mostafiz, Hwal-Su Hwang, Narayan Bhusal, Kyeong-Yeoll Lee

**Affiliations:** 1 Division of Applied Biosciences, College of Agriculture and Life Sciences, Kyungpook National University, Daegu 41566, Republic of Korea; 2 Department of Applied Biosciences, College of Agriculture and Life Sciences, Kyungpook National University, Daegu 41566, Republic of Korea; 3 Department of Agriculture, Forestry, and Biosciences, Seoul National University, Seoul 08826, Republic of Korea; 4 Institute of Plant Medicine, Kyungpook National University, Daegu 41566, Republic of Korea; 5 Sustainable Agriculture Research Center, Kyungpook National University, Gunwi 39061, Republic of Korea

**Keywords:** climate change, integrated pest management, invasive insects, nonlinear models, population dynamics, thermal adaptation

## Abstract

**Simple Summary:**

*Spodoptera frugiperda* is an important agricultural pest of several plants; therefore, a reliable method is required for predicting its emergence in fields. Temperature-dependent development and thermal bio parameters are among approaches that are commonly used to model insect phenology. However, information regarding *S. frugiperda* is limited. In this study, we evaluated the fitness of *S. frugiperda* at various constant temperatures. The results of this study indicated that a temperature range of 28–30 °C was optimal for the fitness of *S. frugiperda.* Among the seven models evaluated in this study, the Shi model best described the relationship between temperature and the development rate of *S. frugiperda*. Estimating thermal thresholds and selecting appropriate models are crucial for effective decision-making regarding *S. frugiperda* control.

**Abstract:**

The fall armyworm *Spodoptera frugiperda* (J.E. Smith) is an economically important pest that recently invaded Africa and Asia; however, information regarding its biological capacity to establish itself in newly invaded environments is largely unknown. We investigated the effects of temperature on the development and survival of the invaded populations of *S. frugiperda* and selected mathematical models to evaluate its development in a new environment. *S. frugiperda* exhibited optimum survival and growth at temperatures between 28 °C and 30 °C. The lower and upper thermal thresholds for the egg-to-adult life cycle were 13.51 °C and 34.13 °C, respectively. We compared seven mathematical models and found that the Shi model was the most suitable for describing the temperature-dependent development rate of *S. frugiperda*. Therefore, the Shi mathematical model may be used to predict both the occurrence of particular developmental stages and the geographic distribution to implement measures for the management of *S. frugiperda* in agricultural fields.

## 1. Introduction

The fall armyworm (FAW) *Spodoptera frugiperda* (J. E. Smith) (Lepidoptera: Noctuidae) is native to the tropical regions of the western hemisphere (Brazil and Argentina) to the southern United States [1]. This species is polyphagous, feeding on more than 350 plants belonging to 76 plant families, including several economically important crops such as maize, rice, and sorghum and various weeds [2]. Recently, the FAW has rapidly invaded Africa and Asia, thereby crucially threatening crop production and food security [1,3,4]. FAW invasion could be attributed to its strong long-distance flight capability, fecundity, and quick adaptability, further modulated by the prevailing meteorological conditions [5,6].

Considering the increasing significance of the FAW as an agricultural pest, it is crucial to understand the factors that affect its fitness and ecology for developing management measures. Temperature is a key abiotic factor in insect growth and development in the environment [7]. Ntiri et al.’s [8] findings suggest that temperature affects resource utilization and intraspecific and interspecific interactions and influences the geographic distribution of ectothermic organisms such as insects [8]. Knowledge regarding the effect of temperature on insect development is critical for modeling their development under environmental conditions.

Mathematical models can be used to evaluate the development rate of an insect pest in response to variations in temperature [9]. A nonlinear curve simulates a lower thermal threshold (T_L_), optimum temperature (T_opt_), and upper thermal threshold (T_H_) [10]. This curve best describes the effect of temperature on the development duration of insects. In this curve, the development rates increase above the lower thermal threshold (T_L_) until they reach the optimum temperature (T_opt_) and then decline to zero at the upper thermal threshold (T_H_) [10]. Because only the highest development rate is attained at T_opt_, certain more elaborated mathematical models also consider parameters that predict the intrinsic optimum temperature [11]. Considering that thermal thresholds can differ among insect species throughout their life cycle, it is essential to select a model that best describes the effect of temperature on the development rate to understand how the insect responds to temperature changes [12].

Based on the best-fit model selection, insect development in environmental conditions can be simulated using temperature time series data [13]. Insect pest control measures can be more successful in some life stages than in others. Hence, inappropriate planning in implementing control measures may lead to failures in pest control [14]. Implementing pest control measures at the ideal time can be cost- and time-effective in the use of pesticides and can thus increase the income for farmers and reduce environmental contamination [15]. Prior planning in implementing the pest control measures approach has been used effectively against various agricultural pests [14,16,17].

Implementing pest control measures depends on mathematical models that reliably predict the development of a pest in environmental conditions. Therefore, models may be used to simulate the phenology of insects, enabling us to set the time of control measures that mimic the presence of vulnerable stages of the pest in the field [18]. Previous studies have investigated the effect of temperature on the development of *S. frugiperda* [19,20,21,22]. However, those studies estimated the temperature thresholds of *S. frugiperda* using linear regression despite the known nonlinear response of ectotherms to temperature. Moreover, few studies on nonlinear models of *S. frugiperda* have either used a single model or focused on environmental parameters other than temperature [23,24,25]. Since its global invasion, the FAW has become a major pest of several crops. Moreover, the effect of temperature on its development has been insufficiently investigated, particularly with respect to the selection of mathematical models for describing this relationship. Therefore, this study aimed to investigate the effect of temperature on the fitness (survival, development time, and growth rate) of the FAW and select mathematical models that describe its development rate.

## 2. Materials and Methods

### 2.1. Colony Maintenance

*S. frugiperda* larvae were collected in August 2019 from a cornfield in Gyeongsan, the Republic of Korea. In our earlier study, this colony was identified as a rice and corn strain based on the mitochondrial cytochrome oxidase subunit I gene (GenBank Accession number MT103342) and nuclear triosephosphate isomerase gene (GenBank Accession number MT89423), respectively [3]. Larvae were maintained at 25 °C ± 1 °C, 60% ± 5% relative humidity (RH) and a 14:10-h light:dark cycle in an insect rearing room according to the protocols outlined in our previous studies [26,27]. Pupae were placed in a plastic cage (40 × 40 × 40 cm^3^) to develop into adults. The adults were then provided 20% sucrose solution within the cage and supplied two corn plant seedlings (~30 days old) in pots as the oviposition substrate. Eggs from the same cluster were used in each experimental setup.

### 2.2. Effect of Temperature on the Fitness of S. frugiperda

The effect of seven constant temperatures on the fitness of *S. frugiperda* was evaluated. From the egg to the adult stage, individuals were placed in temperature-controlled chambers at 15 °C, 20 °C, 25 °C, 28 °C, 30 °C, 32 °C, and 34 °C, 60% ± 5% RH, and a 14 h light:10 h dark photoperiod. To investigate the effect of temperature on embryo development in the FAW, one newly laid egg cluster (~112 eggs/cluster), aged <12 h, was placed in a 100 mL insect breeding dish (SPL Life Sciences, Pocheon-si, Korea) lined with a towel paper. Five replications at each temperature were evaluated. The daily hatching of larvae was recorded to evaluate the period of incubation and the survival rate of eggs.

Newly hatched larvae (*n* = 80 individuals) were separately placed in a 25 mL round disposable plastic vial at each temperature to evaluate the fitness of larval and pupal stages. Larvae were fed on 30-day-old corn leaves (*Zea mays* var. ceratina). The corn leaves were replaced daily, the breeding dish was cleaned, and the number of live individuals and their developmental stages were recorded.

Biological parameters such as survival and development duration, pupal weight, and growth rate were used to determine the effects of temperature on the fitness of the FAW. Sex determination was performed 24 h after pupation. The hardened cuticle and anal genital openings were assessed to distinguish the sex [28,29]. The weight of pupae was determined using a high-precision semi-analytical balance (Ohaus Corporation, Songpa-gu, Seoul 05840, Republic of Korea) 48 h after pupation. The growth rate was determined according to the method reported by Gotthard et al. [30] as follows:

Growth rate = [ln (Pupal weight) − (hatching weight)]/Larval time

### 2.3. Statistical Analysis

#### 2.3.1. Effects of Temperature on the Fitness of *S. frugiperda*

Before statistical analysis, the variables for the homogeneity of group variances and normality were evaluated using Levene’s and Shapiro–Wilk tests, respectively [31]. The development duration at the six constant temperatures was analyzed using the Kruskal–Wallis test (*p* < 0.05). Dunn’s test was used to compare treatments when the differences were statistically significant (*p* < 0.05) [9]. The survival of FAWs at egg, larval, and pupal stages was compared among temperatures using a proportion test [32]. Furthermore, survival curves were plotted for each temperature and compared using the nonparametric Kaplan–Meier method [33]. Pupal weight and growth rate analyses were performed using generalized linear models with the Gaussian distribution family and identity link function. Analyses were conducted using SPSS software, except for the survival analysis, which was performed using the MINITAB software 21.1.

#### 2.3.2. Selection and Evaluation of Mathematical Models

Seven models (six nonlinear and one linear) describing the effects of temperature on the development of insects were fitted to the observed development rate for the egg, larval, and pupal developmental stages of the FAW (Table 1). Three thermal thresholds were considered in this study, including the lower (T_L_) and upper thermal thresholds (T_H_) and optimum temperature (T_opt_). The thermal threshold accuracy of the FAW was evaluated based on both the data from this study and its distribution range. Thermal thresholds estimated beyond the ranges of 8.7–15.0 °C for T_L_, 25.0–35.0 °C for T_opt_, and 33.0–40.0 °C for T_H_ were considered unrealistic [34,35,36,37]. Models that estimated realistic thermal thresholds for the FAW were preferred to those that estimated unrealistic thermal thresholds. The thermal thresholds were estimated using the development rate at each tested temperature. SPSS software and the Levenberg–Marquardt algorithm along with development rate data were used to fit the models, with partial derivatives of the dependent variable guiding estimation with respect to each model parameter [33].

The estimates of the lower thermal threshold (T_L_) and the degree days ((DD); thermal constant *K*) were calculated using the methods described by Campbell et al. [38]. This entailed generating linear regressions to connect the rate of development (y) and temperature (x) for temperature ranges where the relationships are roughly linear. The lower thermal threshold was determined using the x-intercept method (T_L_ = −*a*/*b*) and the constant *K* (the number of heat units required for a species to develop from one stage to the next) obtained via the reciprocal of the slopes (*K* = 1/*b*). When fitting the linear model, data obtained at 15 °C and 34 °C were not used in the analysis because they were outside the linear range between temperature and development rate.

In this study, the number of thermal thresholds estimated using the nonlinear models differed among models. Some models, such as Briere-1, Briere-2, and Shi, estimated T_L_, T_H_, and T_opt,_ directly from the equation or graphically. In contrast, others estimated only T_H_ and T_opt_ (β type and Logan-6) or only T_L_ (linear). The model performance was evaluated using the goodness-of-fit as a statistical criterion and the ability of a single model to correctly estimate temperature thresholds as an additional criterion [9,43,44]. The R square of the regression (R^2^) was used to evaluate the model’s goodness-of-fit. Due to the small sample size, the corrected Akaike information criterion (AICc) was used to evaluate the model’s relative quality as follows:

$$\mathrm{AICc}=nln\left(\frac{RSS}{n}\right)+2k+\left(\frac{2k^{2}+2k}{n-k-1}\right)$$

where *n* denotes the number of treatments, RSS denotes the residual sums of squares, and *k* denotes the number of model parameters. Several researchers currently use AICc to compare the quality of models based on their complexity (number of parameters) and goodness-of-fit, with lower ΔAICc values indicating good performance [45]. Moreover, the ΔAICc was determined as the difference between the score of each model and the lowest AICc in each developmental stage. In this study, ΔAICc values of ≤4.0 indicated similar model performance [46]. The best-fit models in this study were selected based on the accuracy of thermal threshold estimation, the number of thermal thresholds estimated by the model, and the graphical representation of the data in addition to statistical metrics (delta AICc and R^2^).

## 3. Results

### 3.1. Effects of Temperature on the Fitness of S. frugiperda

Temperature had a significant effect on the development time of egg (χ^2^ = 21.58; df = 6; *p* < 0.001), larval (χ^2^ = 25.78; df = 6; *p* < 0.0001), and pupal (χ^2^ = 21.49; df = 5; *p* < 0.001) stages as well as the egg-to-adult life cycle (χ^2^ = 22.70; df = 5; *p* < 0.0001) of the FAW (Table 2).

A negative correlation was observed between temperature and development duration in the range of 15–32 °C (Table 2). The incubation duration varied between 1.33 and 14.25 days at 32 °C and 15 °C, respectively. The larval stage duration varied between 9.11 and 87.42 days at the same temperature range. An increase (but not significant) in the development duration of larvae was observed at 34 °C (13.11 days) compared with that at 32 °C (9.11 days). The pupal stage duration ranged from 6.06 to 20.91 days at 34 °C and 20 °C, respectively. No pupation was recorded at 15 °C. The egg-to-adult life cycle duration ranged from 23.43 to 50.46 days at 32 °C and 20 °C, respectively. The development time of the pupae was affected by temperature (female: χ^2^ = 21.49; df = 5; *p* < 0.001, male: χ^2^ = 21.42; df = 5; *p* < 0.001) but not by sex (Kruskal–Wallis χ^2^ = 2.57; df = 1; *p* = 0.109; Table 2). Moreover, although most larvae developed until six instars, especially at higher temperatures, as many as seven instars were observed for larvae reared at 15 °C and 20 °C, suggesting the temperature dependence of the number of FAW larval instars.

The weight of pupae was significantly affected by temperature (χ^2^ = 242.79; df = 5; *p* < 0.0001) and sex (χ^2^ = 4.11; df = 1; *p* = 0.043; Table 3). Males were heavier than females at all temperatures, except at 20 °C (Table 3).

Moreover, a negative relationship between size and rearing temperature was observed from 30 °C to 34 °C (Table 3). The growth rate was significantly affected by temperature (χ^2^ = 145.61; df = 5; *p* < 0.0001) but not by sex (χ^2^ = 0.301; df = 1; *p* = 0.583). The optimal temperature for the development of the FAW in this study was in the range of 28 °C–30 °C based on pupal weight and growth rate (Table 3).

**Figure 1:**
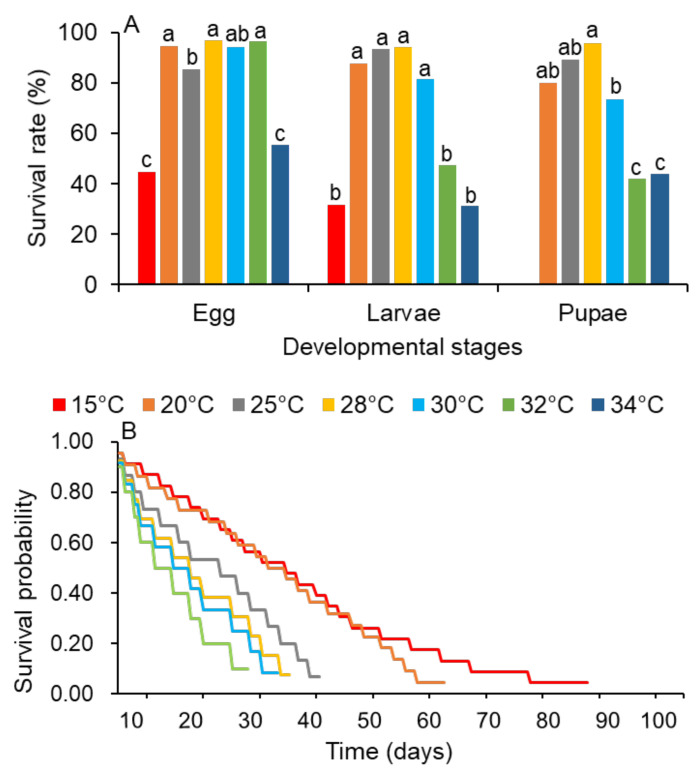
Survival of *S. frugiperda* at different constant temperature regimes. (**A**) Survival rates. (**B**) Survival curves were generated using nonparametric Kaplan–Meier analysis. Survival of egg, larval, and pupal stages was compared among temperatures using a proportion test (*p* < 0.05). Same lowercase letters in each developmental stage denote differences that are not statistically significant at *p* < 0.05.

FAWs developed from the egg to the adult stage at all the tested temperatures, except at 15 °C. The survival of egg (χ^2^ = 12.14; df = 6; *p* < 0.0001), larval (χ^2^ = 42.71; df = 6; *p* < 0.0001), and pupal (χ^2^ = 41.52; df = 5; *p* < 0.0001) stages was significantly affected by temperature (Figure 1A). Kaplan–Meier survival analysis also revealed the significant effect of temperature on the survival rate (Figure 1B).

### 3.2. Selection and Performance of Mathematical Models

The performance of temperature-dependent development models varied based on the developmental stage of *S. frugiperda*. Based on an ΔAICc value, the linear and Taylor were the best-fit models for the egg stage (Table 4). For larvae, the best-fit models were Briere-1 and Briere-2, followed by Taylor. The best performing models for the pupal stage were Shi and Briere-1 and those for the egg-to-adult life cycle were linear, Taylor, and Shi. Conversely, Taylor was the best-fit model for most developmental stages, whereas Shi demonstrated a good fit to the observed data for the pupal stage and egg-to-adult life cycle (Table 4).

**Figure 2:**
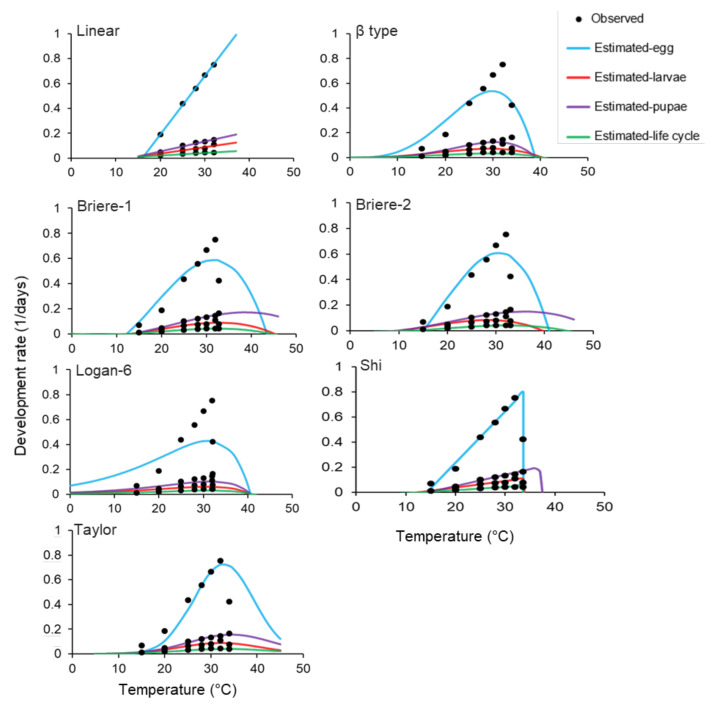
Curves fitted via the mathematical models used to describe the temperature-dependent development rate of *S. frugiperda* for egg, larval, and pupal stages and the egg-to-adult life cycle. While fitting the linear model, data obtained at 15 °C and 34 °C were not used in the analysis because they were outside the linear range between temperature and development rate.

The R^2^ values were in the range 0.70–0.99 when all models were evaluated for the various immature stages of *S. frugiperda* (Table 4). Although most models demonstrated high R^2^ values, their graphical representation revealed significant differences between them (Figure 2). The thermal parameters T_L_, T_H_, and T_opt_ varied widely based on the model evaluated. The T_L_ and T_H_ values in the egg stage varied between 6.50 °C and 14.96 °C and between 34.08 °C and 43.50 °C, respectively. In the larval stage, the T_L_ and T_H_ values were in the range of 8.83–12.88 °C and 34.33–45.31 °C, respectively (Table 5). In the pupal stage, the T_L_ and T_H_ values were in the range of 9.03–14.68 °C and 37.58–54.14 °C, respectively (Table 5). Similarly, the T_L_ and T_H_ values in the egg-to-adult life cycle were in the range of 10.41–13.51 °C and 34.13–44.42 °C, respectively. The T_L_ estimated via linear regression varied between 9.73 °C in the egg-to-adult life cycle and 15.92 °C in the egg stage. The FAWs required 490 DD above the T_L_ to develop from the egg to the adult stage (Table 6). Nevertheless, most models predicted biologically significant parameters, and the estimated values in some cases were unrealistic. For instance, the Briere-1 model estimated very high T_H_ values for all immature stages. Similarly, the Briere-2 model predicted a T_H_ of 51.04 °C and 44.41 °C for the pupal stage and egg-to-adult life cycle, respectively. The Taylor model also predicted a T_L_ value below the expected level for the egg stage (Table 5). However, some models predicted thermal thresholds consistent with the expected values for the FAW based on both its known distribution range and experiments conducted in this study (Table 7). These models included linear, Taylor, Shi, and Briere-2. Considering the goodness-of-fit and the number and accuracy of predicted thermal thresholds, Shi was the most suitable model for the FAW, followed by Taylor (Table 7).

## 4. Discussion

To achieve a better understanding of the phenological change that insect populations go through, it is important to have a clear understanding of how abiotic elements, such as temperature, affect their development rate [47]. This information is especially crucial for invasive species that are expanding their distribution range in invaded areas, such as the FAW. Therefore, understanding the determinants influencing FAW population dynamics is critical for integrated pest management. In this regard, this study could significantly contribute toward understanding the effect of temperature on the development of the FAW.

Our findings demonstrated that temperature significantly affected the development duration and survival rate of the FAW. The effect was observed when the response was compared between individuals at extreme temperatures in this study. For instance, a difference of 27 days was found in the mean development duration between individuals reared at 20 °C and 34 °C. Furthermore, a significant difference in pupal weight was observed between 20 °C and 34 °C, and a lower larval survival rate was observed at 15 °C, 32 °C, and 34 °C. Previous studies have reported similar differences in the development duration of the FAW at different temperatures, wherein lower survival rates of larvae were recorded at high temperatures [21,34]. Interestingly, the number of larval instars (7th instar larvae) increased at 15 °C and 20 °C, which are the lower temperatures at which development was studied, suggesting the biological plasticity of the FAW to survive under adverse conditions [21,29].

Based on biological characteristics such as development time, survival, and pupal weight, our findings imply that the T_opt_ for the FAW is between 28 °C and 30 °C. Our results are consistent with those of previous studies that reported that the most favorable temperature range for FAW development, survival, and reproduction was 27–30 °C [21,22].

Understanding the temperature thresholds of insects is crucial for predicting their potential distribution [48]. In this study, the lower threshold temperature for the development of the egg was estimated at 15.92 °C, which was in the range (15.6–18.3 °C) reported by Barfield et al. [49] but was higher than the threshold temperature of 12.1 °C reported by Prasad et al. [21], 13.01 °C reported by Du Plessis et al. [22], and 12.69 °C reported by Ali et al. [34]. Knowledge regarding the thermal requirements of an insect can help interpret its current geographical distribution and predict its future distribution [50]. Each developmental stage has specific temperature requirements for survival and development in a particular environment [48]. This study demonstrated that the DD requirements for the larvae and pupae of FAW were 193.80 and 124.70, respectively, which were lower than those (204 and 150, respectively) reported by Du Plessis et al. [22]. According to Ali et al. [34], these differences in DD may be attributed to the different larval diets used in the two investigations.

The recent invasion in continents with diverse climatic conditions, such as Asia and sub-Saharan Africa, indicates that the FAW is regularly facing temperatures that are probably outside the linear range of the relationship between temperature and development rate [1,3]. Therefore, linear models become unrealistic to predict the temperature-dependent development rate, which may then necessitate the use of nonlinear models to estimate the development rate of the FAW. In this study, the nonlinear models demonstrated significant variability in their ability to characterize the correlation between temperature and *S. frugiperda* growth rate. In this particular investigation, the level of “goodness-of-fit” was considered alongside the accuracy of the projected thermal thresholds to select the most effective models. The best-fit model in this investigation generated valid estimations of thermal thresholds. The multiple criteria selection method showed that among the seven models assessed, Shi and Taylor can be used to describe the temperature-dependent development of the FAW. Based on the accuracy of the thermal threshold estimation, Shi was considered the best model because it fitted well in all developmental stages. Previous studies have also demonstrated that the Shi model can accurately describe the relationship between temperature and development rate in other lepidopteran species [9,44,51].

We observed differences in the predicted thermal thresholds between the models as well as between the developmental stages. For instance, the T_L_ estimated by the Shi model for the larval stage was 12.78 °C, compared with 8.83 °C and 9.94 °C estimated by the Taylor and Briere-2 models, respectively. For the egg-to-adult life cycle, a difference of 3.1 °C was observed between the T_L_ estimated using the Shi and Taylor models. Interestingly, linear regression estimated a T_L_ value similar to those estimated via the Shi and Briere-1 models but higher than that estimated via the Taylor and Briere-2 models for the larval stage.

The best-fit model obtained in this study can be used in integrated pest management to set a good time to implement management measures. Previously, the same method was used in the management of the oriental fruit moth *Grapholita molesta* (Busck, 1916) (Lepidoptera: Tortricidae) in orchards in North Carolina, the U.S.A. The method contributed to a decrease in pesticide use and decreased the contamination of the environment [52]. Similarly, the findings of this study can be applied to simulate FAW development in the field and provide information on efficient and effective methods for applying control measures. The best-fit model can be used to determine the ideal time to implement control measures as well as assess the effects of climate change on pest voltinism [53]. For example, in a study by Santos et al. [53], the nonlinear model (Lactin-2) predicted a higher number of generations in warmer regions than in colder regions for the small tomato borer *Neoleucinodes elegantalis* (Guenée, 1854) (Lepidoptera: Crambidae). In another study, the nonlinear model predicted a decrease of up to 33.1% in the number of generations of *S. cosmioides* in warmer regions where temperatures often exceeded the optimum temperature required for the species’ survival [54]. The application of mathematical models in pest management might encounter several limitations, including the prediction accuracy of the life stage of the insect in the field and ambiguity in selecting a suitable date to start estimating the development rates [55]. Another potential limitation is associated with the practical application of the model for different crops. In this study, the best-fit model was selected based on development data obtained using the corn plant as the host and only seven mathematical models. Further experiments in different host plants of *S. frugiperda* and evaluation of a greater number number of temperature-dependent development models are essential.

## 5. Conclusions

Temperature had a significant impact on the development and survival of the FAW. The biological factors evaluated in this study indicated that a temperature range of 28 °C–30 °C was optimal for FAW fitness. Among the seven models evaluated in our study, Shi best described the relationship between temperature and the development rate of the FAW. The thermal thresholds estimated via the model suggested that the FAW can undergo development in a broad range of temperatures, which explains the invasion and occurrence of this species in areas with diverse climatic conditions, including tropical, subtropical, and temperate regions. Finally, our study contributes to a better understanding of how temperature affects FAW development. The selected mathematical model will help elucidate the best time to implement pest control measures against this important agricultural pest.

## Figures and Tables

**Table 1:** Mathematical models used to describe the temperature-dependent development rate of *Spodoptera frugiperda*.

Model	Function	Reference
linear	D(T) = a + bT	[38]
β type	D(T) = ρ.(aT/10).(T/10)^β^	[39]
Briere-1	D(T) = aT(T − T_L_)(T_H_ − T)^1/2^	[40]
Briere-2	D(T) = aT(T − T_L_)(T_H_ − T)^1/m^	[40]
Shi	D(T) = m(T − T_L_(1− $e^{k\left(T-TH\right)}$)	[41]
Logan-6	D(T) = Ψ($e^{\rho T}-e^{\left(\rho TH-\frac{TH.T}{\Delta }\right)}$	[10]
Taylor	D(T) = Rm.EXP − 0.5(T − T_opt_)^2^	[42]

D(T) is the development rate at temperature T (°C), T_L_ the lower temperature threshold, T_H_ the upper-temperature threshold, and T_opt_ the optimum temperature for development. The remaining parameters are fitted coefficients.

**Table 2:** Development duration (in days [mean ± SE]) of *Spodoptera frugiperda* at different constant temperature regimes.

Temperature (°C)	N *	Egg †	Larvae	Pupae			Egg-to-Adult
				Male	Female	Mean	
15	80	14.25 ± 1.31 ^a^	87.42 ± 2.68 ^a^	-	-	-	-
20	87	5.33 ± 0.88 ^b^	25.41 ± 0.93 ^ab^	21.85 ± 0.45 ^a^	19.6 ± 1.43 ^a^	20.91 ± 0.70 ^a^	50.46 ± 1.71 ^a^
25	70	2.29 ± 0.17 ^bc^	15.37 ± 0.84 ^ab^	10.25 ± 0.25 ^ab^	9.57 ± 0.12 ^ab^	9.72 ± 0.125 ^ab^	31.88 ± 0.25 ^ab^
28	103	2.00 ± 0.00 ^c^	13.61 ± 0.22 ^bc^	8.75 ± 0.18 ^b^	7.55 ± 0.16 ^b^	8.15 ± 0.15 ^b^	25.22 ± 0.58 ^abc^
30	90	1.50 ± 0.28 ^c^	12.64 ± 0.31 ^c^	7.87 ± 0.39 ^bc^	7.40 ± 0.63 ^bc^	7.58 ± 0.57 ^bc^	23.44 ±1.27 ^bc^
32	80	1.33 ± 0.33 ^c^	9.11 ± 0.30 ^c^	7.12 ± 0.21 ^bc^	6.62 ± 0.17 ^bc^	6.87 ± 0.14 ^bc^	23.43 ± 0.38 ^c^
34	80	2.36 ± 0.06 ^c^	13.11 ± 0.34 ^c^	6.83 ± 0.40 ^c^	5.81 ± 0.23 ^c^	6.06 ± 0.21 ^c^	23.87 ± 0.42 ^c^

* Number of individuals of first instar larvae; † five egg clusters (approximately 100 egg/mass); -no larvae pupated at 15 °C. Means within a column followed by the same letter are not significantly different (Krukal–Wallis test followed by Dunn’s multiple comparison test, *p* < 0.05).

**Table 3:** Pupal weight (mean ± SE) and growth rate of *Spodoptera frugiperda* reared at different constant temperature regimes.

Temperature (°C)	Pupal Weight (mg)		Growth Rate (mg/Day)	
	Male	Female	Male	Female
20	194.42 ± 3.74 ^aB^ (20) ^†^	202.12 ± 6.15 ^aA^ (23) ^†^	8.31 ± 0.31 ^c^	9.26 ± 0.87 ^d^
25	142.98 ± 9.27 ^bA^ (13)	139.53 ± 4.10 ^bB^ (18)	8.32 ± 0.31 ^c^	9.51 ± 0.71 ^d^
28	193.96 ± 10.88 ^aA^ (52)	192.06 ± 6.30 ^aB^ (48)	14.59 ± 0.77 ^a^	13.87 ± 0.38 ^a^
30	167.26 ± 7.90 ^bA^ (25)	150.24 ± 5.73 ^bB^ (33)	13.13 ± 1.25 ^a^	14.68 ± 1.4 ^a^
32	126.43 ± 4.65 ^cA^ (16)	120.54 ± 4.72 ^cB^ (13)	12.11 ± 0.98 ^b^	10.94 ± 0.67 ^b^
34	127.35 ± 14.95 ^cA^ (16)	117.80 ± 6.05 ^cB^ (15)	10.79 ± 0.97 ^b^	9.75 ± 0.72 ^c^

† Sample sizes; means within a column followed by the same lowercase within column and uppercase letters within rows are not significantly different (GLM with Gaussian distribution followed by LSD multiple comparison test, *p* < 0.05).

**Table 4:** Mathematical models evaluation based on the R square (R^2^) and the corrected Akaike information criterion (AICc).

Model	Egg		Larvae		Pupae		Egg–Adult	
	R^2^	∆AICc	R^2^	∆AICc	R^2^	∆AICc	R^2^	∆AICc
Linear	0.99	0.00	0.91	8.99	0.97	7.13	0.96	0.00
β type	0.81	8.29	0.71	8.91	0.86	20.93	0.74	11.29
Briere-1	0.91	4.46	0.95	0.00	1.00	2.89	0.94	4.17
Briere-2	0.86	6.68	0.95	0.00	0.98	9.91	0.94	4.17
Shi	0.88	5.96	0.86	5.21	0.99	0.00	0.95	0.99
Logan	0.89	5.54	0.88	4.33	0.93	17.59	0.91	5.97
Taylor	0.96	0.25	0.95	0.10	0.99	8.93	0.97	0.13

ΔAICc values in bold represent the models with similar performance (ΔAICc < 4).

**Table 5:** Mathematical model with parameter values of different life stages of *Spodoptera frugiperda*.

Model	Egg	Larvae	Pupae	Egg–Adult
**Linear**				
a (10^−2^)	0.00	−6.60	−10.64	−1.99
b (10^−2^)	4.70	0.52	0.80	0.20
T_L_	15.92	12.78	13.26	9.73
**β-type**				
ρ (10^−3^)	15.88	3.70	2.72	1.67
a	3.89	4.04	3.90	4.04
b	3.31	2.64	3.58	2.71
T_H_	38.86	40.36	39.01	40.33
T_opt_	30.28	30.10	31.09	30.13
**Briere-1**				
a (10^−5^)	16.12	2.15	2.29	1.14
T_L_	12.23	12.88	13.47	13.08
T_H_	43.50	45.31	54.14	44.42
T_opt_	33.82	33.19	33.58	32.77
**Briere-2**				
a (10^−6^)	89.54	45.39	32.38	24.80
m	7.27	3.19	3.04	4.35
T_L_	14.96	9.94	9.77	13.09
T_H_	40.90	39.53	51.04	44.41
T_opt_	31.21	30.35	36.74	32.99
**Shi**				
K	14.16	3.51	2.60	12.79
m (10^−3^)	41.00	5.16	9.03	2.56
T_L_	14.27	12.78	14.68	13.51
T_H_	34.08	34.33	37.58	34.13
T_opt_	33.91	33.62	36.35	33.95
**Logan-6**				
Ρ	0.10	0.10	0.11	0.10
Δ	9.12	9.56	8.96	9.07
Ψ	0.22	0.18	0.13	0.01
T_H_	40.61	40.63	40.71	41.09
T_opt_	32.12	32.00	32.43	32.13
**Taylor**				
Rm (10^−2^)	72.49	8.87	15.75	4.18
T_L_	6.50	8.83	9.03	10.41
T_opt_	32.74	32.04	34.38	33.38

Thermal thresholds were estimated using the Levenberg–Marquardt algorithm along with development rate data in SPSS and solver in MS excel.

**Table 6:** Regression of development rates, developmental lower thresholds (T_L_), and total thermal constants (K) (degree-days) of *Spodoptera frugiperda* at five constant temperatures.

Life Stage	Regression Parameters					
	Equation	Slope ± SEM	Intercept	R^2^	K (Degree-Days)	T_L_ (°C)
Egg	Y = 0.04696 * X − 0.7474	0.04696 ± 0.0009017	−0.7474 ± 0.02464	0.999	21.29	15.92
Total larval	Y = 0.00516 * X − 0.06596	0.00516 ± 0.0009558	−0.06596 ± 0.02612	0.907	193.80	12.78
Pupa	Y = 0.00802 * X − 0.1064	0.00802 ± 0.0007856	−0.1064 ± 0.02146	0.972	124.70	13.27
Egg–adult	Y = 0.002041 * X − 0.01986	0.002041 ± 0.0002483	−0.01986 ± 0.006786	0.958	490.00	9.73

Y represents the development rate (inverse of development time = 1/D) at temperature X in the formula Y = a + bX. The lower thermal threshold was determined using the x-intercept method (*T_L_* = −*a*/*b*) and the constant *K* (the number of heat units required) obtained via the reciprocal of the slopes (*K* = 1/*b*).

**Table 7:** Thermal thresholds estimated via each model and their accuracy based on the development duration and known distribution range of *Spodoptera frugiperda*.

Model	Number of Estimated Thermal Threshold	Accuracy ª		
		T_L_	T_opt_	T_H_
Linear	1	+	*	*
β type	2	*	+	−
Briere-1	3	+	+	−
Briere-2	3	+	+	−
Lactine-2	3	−	+	−
Shi	3	+	+	+
Logan-6	2	*	+	−
Taylor	2	+	+	*

T_L_ and T_H_ are the lower and upper thermal thresholds, respectively, and T_opt_ is the optimum temperature. ª: +, yes; −, no; *, parameter not estimated by the model.

## Data Availability

The data presented in this study are available within the paper.
